# Fear of Cancer Recurrence and Psychological Symptom Burden in Breast Cancer Survivors Without Evidence of Active Disease: A Cross-Sectional Real-World Study

**DOI:** 10.3390/healthcare14162665

**Published:** 2026-08-21

**Authors:** Salih Karatlı, Safiye Kübra Çetindağ Karatlı, Selahattin Çelik, Tülay Eren, Doğan Yazılıtaş

**Affiliations:** 1 Department of Medical Oncology, Ankara Etlik City Hospital, 06010 Ankara, Türkiye; 2 Department of Family Medicine, Ankara Bilkent City Hospital, 06760 Ankara, Türkiye; 3 Department of Family Medicine, Ankara Yıldırım Beyazıt University, 06760 Ankara, Türkiye

**Keywords:** breast cancer, fear of cancer recurrence, survivorship, anxiety, depression

## Abstract

**Objective**: This study assessed the prevalence of fear of cancer recurrence (FCR) and its clinical, treatment-related, and psychological correlates in breast cancer survivors without active disease. **Materials and Methods**: This cross-sectional study included 200 breast cancer survivors after curative treatment. FCR was assessed using the Fear of Cancer Recurrence Inventory–Short Form (FCRI-SF), with scores ≥ 13 indicating clinically significant FCR. Hospital Anxiety and Depression Scale-Anxiety (HADS-A) and Depression (HADS-D) scores ≥ 8 indicated clinically significant symptoms. Associations with FCR were analyzed using logistic regression, with HADS-A and HADS-D evaluated in separate multivariable models because of their potential overlap. **Results**: The mean age was 55.6 ± 9.5 years; clinically significant FCR, anxiety, and depression occurred in 38.0%, 32.5%, and 38.0%, respectively. In univariable analysis, younger age, stage III disease, mastectomy, chemotherapy history, and high HADS-A and HADS-D were associated with FCR. In the HADS-A model, chemotherapy history (adjusted OR: 5.841; *p* = 0.006) and high HADS-A (adjusted OR: 7.828; *p* < 0.001) were independently associated with FCR, whereas increasing age was protective (adjusted OR: 0.855; *p* < 0.001). In the HADS-D model, mastectomy (adjusted OR: 6.265; *p* = 0.002), chemotherapy history (adjusted OR: 6.941; *p* = 0.001), and high HADS-D (adjusted OR: 14.343; *p* < 0.001) were independently associated with FCR, whereas increasing age was protective (adjusted OR: 0.836; *p* < 0.001). **Conclusions**: FCR is a major psychosocial concern among breast cancer survivors, particularly in younger patients, those with chemotherapy history, and those with greater psychological symptom burden. The association with mastectomy should also be considered, particularly in patients with depressive symptoms.

## 1. Introduction

Breast cancer is one of the most common malignancies among women, and substantial improvements in survival have been achieved through advances in early diagnosis and multimodal treatment approaches. As a result, a considerable proportion of patients diagnosed with breast cancer achieve long-term survival and are followed as cancer survivors after treatment. Increasing survival has also heightened the importance of physical, psychological, and social problems that may emerge during the post-treatment period [1,2,3].

One of the most frequently reported psychosocial problems among cancer survivors is fear of cancer recurrence (FCR). FCR is defined as persistent, unwanted, and distressing concern, anxiety, or fear that cancer may recur or progress. This condition may adversely affect patients’ quality of life, sleep patterns, social relationships, work life, and overall psychological well-being. It is also closely associated with symptoms of anxiety and depression and may constitute a clinically significant psychological burden in some patients [4,5,6]. Recent systematic reviews have further highlighted the clinical relevance of FCR among breast cancer survivors and suggest that targeted psychological interventions, including cognitive behavioral and mindfulness-based approaches, may reduce FCR in selected survivor populations [7].

The frequency of FCR among breast cancer survivors varies across studies, and clinically significant FCR has been reported in a substantial proportion of patients. Factors associated with FCR commonly include younger age, advanced disease stage, history of mastectomy, receipt of chemotherapy, symptoms of anxiety and depression, and low social support. However, the frequency of FCR and its associated factors may differ across populations because of variations in cultural characteristics, socioeconomic status, access to healthcare, and follow-up practices [8,9,10].

Although the association of FCR with anxiety and depression has been well documented, important gaps remain regarding how psychological symptoms interact with clinical and treatment-related characteristics in breast cancer survivors who have completed curative treatment and have no evidence of active disease. Moreover, although the full FCRI has undergone validity and reliability testing in Turkish cancer populations [11], real-world data from Türkiye examining these factors simultaneously in routine oncology practice remain limited. Therefore, the distinctive contribution of the present study is the integrated evaluation of sociodemographic, clinicopathological, treatment-related, and psychological factors associated with clinically significant FCR in a real-world cohort of breast cancer survivors without active disease. By identifying factors independently associated with clinically significant FCR, this study may also help define survivor subgroups who could benefit from more focused psychosocial assessment during routine follow-up.

Accordingly, this study aimed to determine the frequency of clinically significant FCR among breast cancer survivors who had completed curative treatment and had no evidence of active disease, and to evaluate its independent associations with sociodemographic, clinicopathological, treatment-related, and psychological factors.

## 2. Materials and Methods

### 2.1. Study Design and Patient Population

This study was designed as a cross-sectional, questionnaire-based real-world study. The study was conducted at the Department of Medical Oncology, Ankara Etlik City Hospital, between 18 June and 18 July 2026.

An a priori power analysis was performed to determine the required sample size. Assuming a two-sided significance level of 0.05, a statistical power of 80%, and an anticipated small-to-moderate effect size, the minimum required sample size was estimated to be approximately 190 participants. To account for possible incomplete questionnaires or missing clinical data, the target sample size was increased to 200 participants.

Breast cancer survivors who had undergone surgery for breast cancer, completed curative-intent neoadjuvant and/or adjuvant chemotherapy and/or radiotherapy, and had no evidence of active disease at the time of assessment were included.

Patients who agreed to participate completed the questionnaires in a face-to-face, interviewer-administered format conducted by a trained investigator. The questionnaire included sociodemographic characteristics, clinicopathological features, treatment-related variables, and psychological assessment results. Age, menopausal status, educational level, income status, smoking history, disease stage at diagnosis, molecular subtype, type of surgery, history of chemotherapy and radiotherapy, hormone therapy use and duration, history of targeted therapy, and remission duration were recorded.

### 2.2. Inclusion and Exclusion Criteria

#### 2.2.1. Inclusion Criteria

The inclusion criteria were as follows:

1. Being 18 years of age or older.
2. Having a histopathologically confirmed diagnosis of breast cancer.
3. Having undergone surgical treatment for breast cancer.
4. Having completed curative-intent neoadjuvant and/or adjuvant chemotherapy and/or radiotherapy.
5. Having no evidence of active recurrence or metastatic disease at the time of assessment.
6. Having sufficient cognitive and communicative ability to understand and answer the questionnaire items.
7. Agreeing to participate in the study and providing informed consent.

#### 2.2.2. Exclusion Criteria

Patients with any of the following characteristics were excluded:

1. Evidence of active recurrence or metastatic disease at the time of assessment.
2. Cognitive impairment, dementia, or severe communication problems preventing completion of the questionnaire forms.
3. Active psychotic disorder, severe major depression, acute psychiatric crisis, or any severe psychiatric condition that could affect participation in the study or the reliability of scale responses.
4. Refusal to participate in the study or failure to provide informed consent.
5. Missing core clinical or scale data preventing analysis.
6. History of a second malignancy other than breast cancer.

### 2.3. Data Collection and Variable Definitions

Data were collected using a standardized case report and questionnaire form developed by the investigators. Remission duration was calculated as the time from completion of the curative treatment process to the date of questionnaire administration and was recorded in months.

Educational level was analyzed in two groups. Patients who were illiterate, literate without formal schooling, or primary school graduates were categorized as having a low educational level, whereas patients who had completed high school or university were categorized as having a higher educational level. Income status was recorded according to the patient’s self-report as income lower than expenses, income equal to expenses, or income higher than expenses. Molecular subtypes were categorized as HR-positive/HER2-negative, HER2-positive, and triple-negative. The HER2-positive group included tumors with HER2 positivity irrespective of hormone receptor status, whereas triple-negative tumors were defined as ER-negative, PR-negative, and HER2-negative.

### 2.4. Assessment of Fear of Cancer Recurrence

Fear of cancer recurrence was assessed using the 9-item Fear of Cancer Recurrence Inventory–Short Form (FCRI-SF), which corresponds to the Severity domain of the original FCRI [12]. The full FCRI has been translated and validated in Turkish cancer populations and has demonstrated satisfactory psychometric properties (overall Cronbach’s α = 0.94) [11]. Although a separate formal validation study specifically evaluating the 9-item FCRI-SF as an independent short-form instrument in Turkish populations has not been identified, the FCRI-SF is derived directly from the Severity domain of the original FCRI. In the present study, these nine Severity-domain items were administered in a face-to-face, interviewer-administered format. Each item is scored from 0 to 4, with higher total scores indicating higher levels of FCR. An FCRI-SF score of ≥13 was used as the predefined threshold for clinically significant FCR, consistent with previous studies in breast cancer populations [13,14]. This threshold was selected to maintain comparability with the existing breast cancer literature; however, it has not been specifically validated as a population-specific diagnostic cut-off in Turkish breast cancer survivors.

### 2.5. Assessment of Anxiety and Depression

Anxiety and depression were assessed using the validated Turkish version of the Hospital Anxiety and Depression Scale (HADS). HADS is a two-subscale instrument used to screen for symptoms of anxiety and depression. The HADS-Anxiety subscale (HADS-A) and HADS-Depression subscale (HADS-D) scores were recorded separately. In accordance with the literature, a score of ≥8 on the HADS-A or HADS-D subscale was accepted as the threshold for clinically significant anxiety or depression symptoms [15,16].

### 2.6. Ethical Approval

The study was approved by the Ankara Etlik City Hospital Scientific Research Ethics Committee on 17 June 2026, with approval number AEŞH-BADEK1-2026-460. The study was conducted in accordance with the principles of the Declaration of Helsinki. All participants were informed about the study, and informed consent was obtained from patients who agreed to participate.

### 2.7. Statistical Analysis

Statistical analysis was performed using IBM SPSS Statistics for Windows, version 26.0 (IBM Corp., Armonk, NY, USA). The normality of continuous variables was assessed using visual methods and appropriate statistical tests. Normally distributed continuous variables were expressed as mean ± standard deviation, whereas non-normally distributed variables were presented as median and minimum–maximum values. Categorical variables were presented as numbers and percentages. Patients with missing core questionnaire or clinical data required for the primary analyses were excluded from the final analytic cohort, and no missing-data imputation was performed.

Patients with and without clinically significant FCR were compared in terms of sociodemographic, clinicopathological, and treatment-related variables. Categorical variables were compared using the chi-square test or Fisher’s exact test. Continuous variables were compared using Student’s *t*-test or the Mann–Whitney U test according to distribution characteristics.

FCRI-SF, HADS-A, and HADS-D scores were dichotomized using predefined cut-off values to facilitate clinical interpretation and to identify patients with clinically significant psychological symptoms.

Logistic regression analysis was performed to evaluate factors associated with clinically significant FCR. Clinically significant FCR was considered the dependent variable. Variables with *p* < 0.10 in univariable logistic regression analyses and variables considered clinically important were included in the multivariable logistic regression model. Because HADS-A and HADS-D represent conceptually related and potentially overlapping dimensions of psychological distress, they were not entered simultaneously into the same multivariable model. Instead, two parallel multivariable logistic regression models were constructed to evaluate their individual associations with clinically significant FCR while maintaining the interpretability of the clinical and treatment-related covariates. Model 1 included HADS-A, whereas Model 2 included HADS-D, with the remaining covariates kept consistent across both models. Because age and menopausal status were closely related, both variables were not entered simultaneously into the multivariable models. Age was retained in the primary models because it was analyzed as a continuous variable and therefore preserved more information. As a sensitivity analysis, both multivariable models were repeated by replacing age with menopausal status. Results of univariable analyses were presented as odds ratios (ORs), whereas results of multivariable analyses were presented as adjusted odds ratios (adjusted ORs), with 95% confidence intervals (CIs). A *p* value of <0.05 was considered statistically significant. To ensure the stability and validity of the multivariable logistic regression models, multicollinearity among the independent variables (age, disease stage, type of surgery, chemotherapy history, and HADS scores) was formally evaluated by calculating the Tolerance and Variance Inflation Factor (VIF) using a linear regression diagnostic framework. A VIF threshold of >5.0 was considered indicative of significant multicollinearity. Model calibration was evaluated using the Hosmer–Lemeshow goodness-of-fit test, explanatory performance was summarized using Nagelkerke’s pseudo-R^2^, and discriminative performance was assessed using receiver operating characteristic analysis and the area under the curve (AUC).

## 3. Results

**Figure 1:**
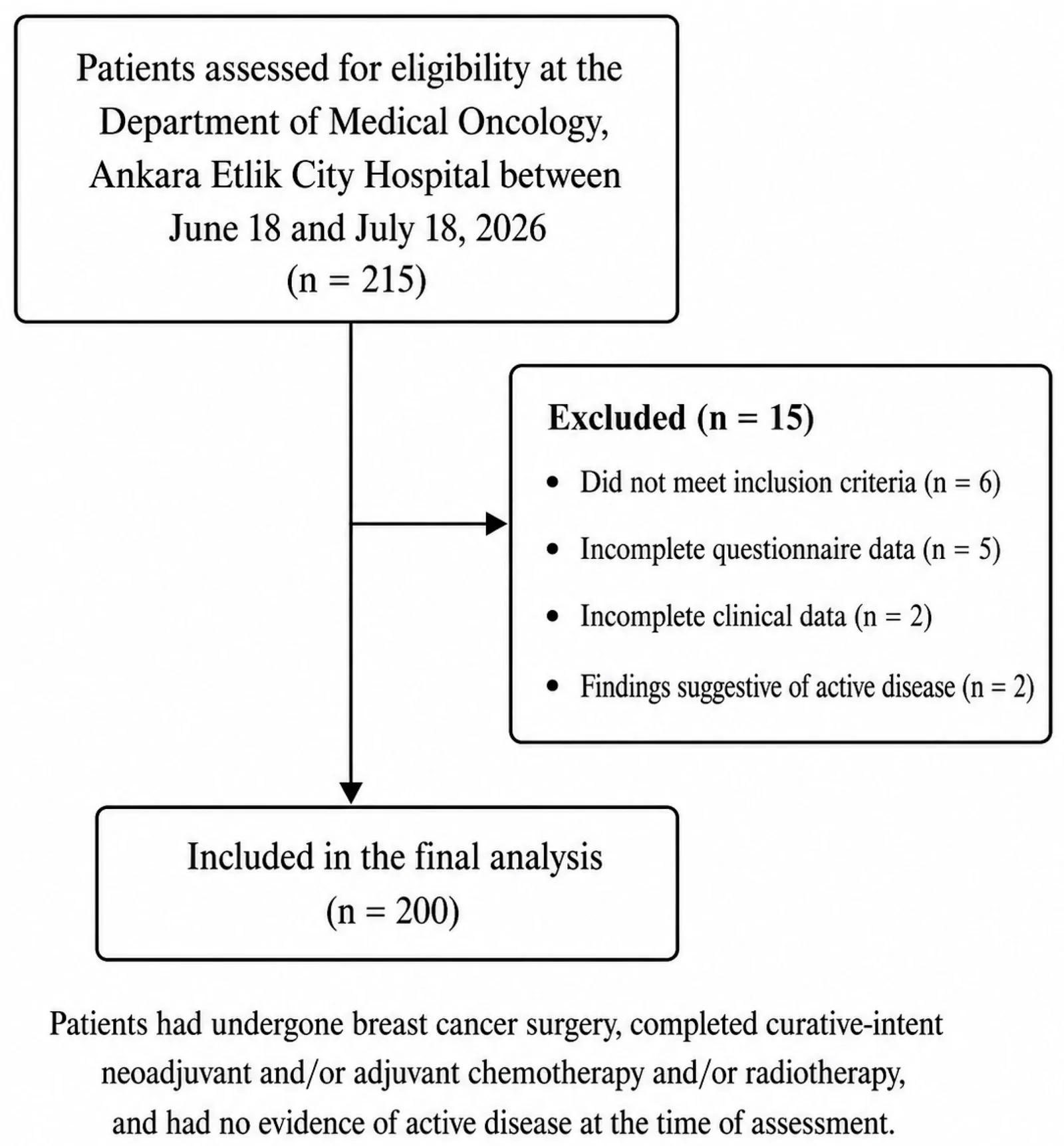
Flow diagram of patient selection and inclusion in the final analysis.

During the study period, 215 patients were assessed for eligibility. Fifteen patients were excluded because they did not meet the inclusion criteria, had incomplete questionnaires or clinical data, or had findings suggestive of active disease. Finally, 200 patients were included in the final analysis, corresponding to an overall inclusion rate of 93.0%. The patient selection process is shown in Figure 1.

The mean age of the patients was 55.60 ± 9.51 years, with a median age of 56 years (range: 38–73). The majority of participants were postmenopausal (78.0%) and had a low educational level (69.5%). Overall, 34.5% of the patients reported that their income was lower than their expenses, and 20.0% had a history of smoking. Stage III disease was the most common disease stage at diagnosis (57.0%). Regarding molecular subtype, hormone receptor-positive/HER2-negative disease was the most frequent subtype (61.0%), followed by HER2-positive (27.0%) and triple-negative (12.0%) disease. Breast-conserving surgery had been performed in 64.0% of patients, whereas 36.0% had undergone mastectomy. A history of chemotherapy and radiotherapy was present in 70.0% and 76.5% of patients, respectively. The median remission duration was 24 months (range: 6–96). The baseline demographic, clinicopathological, and treatment-related characteristics of the patients are presented in Table 1.

Clinically significant FCR was detected in 76 patients (38.0%). Clinically significant anxiety and depression symptoms were observed in 65 (32.5%) and 76 (38.0%) patients, respectively. Findings related to FCR, HADS-A, and HADS-D are presented in Table 2.

In comparisons according to clinically significant FCR status, pre-/perimenopausal status (*p* < 0.001), disease stage (*p* < 0.001), history of mastectomy (*p* = 0.009), history of chemotherapy (*p* = 0.031), and clinically significant anxiety/depression symptoms (*p* < 0.001 for both) were significantly associated with FCR. The frequency of FCR was higher particularly among pre-/perimenopausal patients, those with stage III disease, patients who had undergone mastectomy, those with a history of chemotherapy, and patients with HADS-A or HADS-D scores ≥ 8. In contrast, no significant differences were observed according to educational level, income status, remission duration, smoking history, molecular subtype, or history of radiotherapy. Comparisons of patient characteristics according to clinically significant FCR status are presented in Table 3.

In the univariable logistic regression analysis, in which clinically significant FCR was considered the dependent variable, increasing age (OR: 0.864; 95% CI: 0.827–0.903; *p* < 0.001) and postmenopausal status (OR: 0.063; 95% CI: 0.026–0.154; *p* < 0.001) were associated with a lower likelihood of FCR. Stage III disease (OR: 6.389; 95% CI: 2.769–14.740; *p* < 0.001), history of mastectomy (OR: 2.200; 95% CI: 1.214–3.986; *p* = 0.009), history of chemotherapy (OR: 2.062; 95% CI: 1.063–4.002; *p* = 0.032), clinically significant anxiety symptoms (HADS-A ≥ 8; OR: 5.425; 95% CI: 2.861–10.287; *p* < 0.001), and clinically significant depression symptoms (HADS-D ≥ 8; OR: 3.953; 95% CI: 2.153–7.258; *p* < 0.001) were associated with a higher likelihood of FCR (Table 4).

In multivariable logistic regression analyses, HADS-A and HADS-D were evaluated in two separate models because of the potential overlap between anxiety and depression symptoms. In Model 1, which included HADS-A, increasing age was associated with a lower likelihood of clinically significant FCR (adjusted OR: 0.855; 95% CI: 0.807–0.906; *p* < 0.001), whereas chemotherapy history (adjusted OR: 5.841; 95% CI: 1.680–20.309; *p* = 0.006) and clinically significant anxiety symptoms (adjusted OR: 7.828; 95% CI: 3.200–19.154; *p* < 0.001) were independently associated with higher FCR. Disease stage and mastectomy did not retain independent significance in this model (Table 5).

In Model 2, which included HADS-D, increasing age remained independently associated with a lower likelihood of FCR (adjusted OR: 0.836; 95% CI: 0.787–0.887; *p* < 0.001). Mastectomy (adjusted OR: 6.265; 95% CI: 1.987–19.749; *p* = 0.002), chemotherapy history (adjusted OR: 6.941; 95% CI: 2.109–22.847; *p* = 0.001), and clinically significant depression symptoms (adjusted OR: 14.343; 95% CI: 4.398–46.779; *p* < 0.001) were independently associated with higher FCR. Disease stage was not independently associated with FCR (Table 6).

Across both models, younger age, chemotherapy history, and psychological symptom burden were consistently associated with clinically significant FCR, whereas mastectomy remained independently significant only in the HADS-D model. Additionally, collinearity diagnostics revealed that VIF values ranged from 1.098 to 1.391 in Model 1 and from 1.094 to 1.622 in Model 2, indicating no evidence of problematic multicollinearity in either model.

Sensitivity analyses replacing age with menopausal status are presented in Supplementary Tables S1 and S2. In the model including HADS-A, postmenopausal status was independently associated with lower odds of FCR (adjusted OR: 0.083; 95% CI: 0.029–0.237; *p* < 0.001), while chemotherapy history (adjusted OR: 3.341; 95% CI: 1.249–8.940; *p* = 0.016) and clinically significant anxiety symptoms (adjusted OR: 4.878; 95% CI: 2.197–10.827; *p* < 0.001) remained significant. In the model including HADS-D, postmenopausal status was similarly associated with lower odds of FCR (adjusted OR: 0.049; 95% CI: 0.016–0.149; *p* < 0.001), while mastectomy (adjusted OR: 4.496; 95% CI: 1.657–12.198; *p* = 0.003), chemotherapy history (adjusted OR: 4.423; 95% CI: 1.582–12.372; *p* = 0.005), and clinically significant depression symptoms (adjusted OR: 7.541; 95% CI: 2.849–19.961; *p* < 0.001) remained significant.

Model 1 had a Nagelkerke pseudo-R^2^ of 0.625. The Hosmer–Lemeshow goodness-of-fit test was significant (χ^2^ = 17.451, df = 8, *p* = 0.026), while the AUC was 0.919, indicating strong discriminative performance. Model 2 had a Nagelkerke pseudo-R^2^ of 0.552. The Hosmer–Lemeshow goodness-of-fit test was also significant (χ^2^ = 45.029, df = 8, *p* < 0.001), while the AUC was 0.919. These findings indicate strong discriminative ability in both models, although the significant Hosmer–Lemeshow tests suggest that model calibration should be interpreted with caution.

## 4. Discussion

This study demonstrated that FCR is a clinically important psychosocial problem among breast cancer survivors who had completed curative treatment processes and had no evidence of active disease. In two separate multivariable models including HADS-A and HADS-D, younger age, chemotherapy history, and psychological symptom burden were consistently associated with clinically significant FCR. Mastectomy was independently associated with FCR in the model including HADS-D, but not in the model including HADS-A. These findings suggest that FCR cannot be explained solely by biomedical variables such as tumor stage or molecular subtype; rather, it represents a multidimensional survivorship issue that should be evaluated together with treatment experience, age, and psychological symptom burden.

Anxiety and depression symptoms are important psychosocial problems that may persist after completion of treatment in patients with cancer and cancer survivors. Studies evaluating long-term cancer survivors have reported that symptoms of depression and anxiety remain clinically relevant during follow-up [17,18]. Studies focusing specifically on breast cancer also indicate that these symptoms are not limited to the early treatment period and may continue throughout survivorship [19,20]. In the present study, clinically significant anxiety and depression symptoms were observed in a substantial proportion of patients, suggesting that psychological symptom burden persists even among breast cancer survivors without evidence of active disease and is generally consistent with the literature.

FCR is considered one of the major psychosocial burdens reported by patients and survivors across different cancer types. Reviews evaluating FCRI-based studies have emphasized that the prevalence of FCR varies substantially depending on the cut-off value used, making direct comparisons between studies difficult [21]. The fact that FCR does not completely resolve spontaneously over time and may adversely affect quality of life, psychological distress, and healthcare utilization highlights the need for active assessment of this issue during post-treatment follow-up [22,23].

Previous studies have reported that FCR negatively affects health-related quality of life in long-term breast cancer survivors and is not limited to the early post-treatment period [4]. Differences across studies may be explained by variations in the scales and cut-off values used, differences in the timing of post-treatment assessment, cultural characteristics, and levels of social support.

In the present study, remission duration was not significantly associated with clinically significant FCR. This finding is consistent with previous research in breast cancer survivors showing that time since the last treatment did not predict overall fear of recurrence. Van den Beuken-van Everdingen et al. similarly reported that FCR levels were independent of time since the last treatment among breast cancer survivors. However, findings in the literature are not entirely consistent, as some studies have reported higher FCR among survivors assessed closer to primary treatment. These differences may reflect variations in survivor populations, follow-up duration, assessment timing, and FCR measurement instruments [24].

When factors associated with FCR are considered, younger age, treatment intensity, history of mastectomy and chemotherapy, psychological distress, and coping difficulties have been highlighted in the literature [22,23]. In the present study, younger age and history of chemotherapy were consistently associated with clinically significant FCR across both multivariable models. Sensitivity analyses replacing age with menopausal status demonstrated a strong inverse association between postmenopausal status and FCR, suggesting that the observed age-related association may partly reflect the close relationship between age and menopausal status. Mastectomy may be related to FCR through its effects on body image, illness perception, and perceived treatment burden. The observed association between chemotherapy history and FCR should be interpreted cautiously, as chemotherapy may also serve as a surrogate marker of greater disease severity or treatment intensity rather than representing an independent driver of FCR. In addition, the independent association between clinically significant anxiety and depression symptoms and FCR supports the view that FCR is closely related to anxiety and depression, while representing a distinct psychological burden that should be evaluated separately.

Although disease stage was significantly associated with FCR in the univariable analysis, it did not retain independent significance in either multivariable model. Because Stage III disease is clinically associated with a greater likelihood of receiving chemotherapy and undergoing mastectomy, potential multicollinearity among these variables was assessed. The low VIF values indicated no evidence of problematic multicollinearity or substantial model instability. Therefore, the loss of statistical significance for disease stage is unlikely to be explained by a mathematical artifact arising from highly correlated predictors. Instead, the observed association between advanced stage and FCR may be partly explained by treatment-related experiences, including mastectomy and chemotherapy, as well as by psychological symptom burden. These findings suggest that disease stage may be less directly associated with FCR once treatment-related and psychological factors are taken into account. However, because a formal mediation analysis was not performed, this interpretation should be considered hypothesis-generating rather than evidence of a definitive mediating pathway.

From a clinical perspective, these findings support consideration of structured FCR assessment during routine survivorship follow-up. Brief validated screening tools may help identify patients with clinically significant FCR, particularly among younger survivors and those with greater psychological symptom burden or a history of more intensive treatment. Patients with clinically significant FCR or coexisting anxiety/depressive symptoms may benefit from further psychosocial assessment and, when appropriate, referral to psycho-oncology or psychological support services. Evidence-based interventions such as cognitive-behavioral and other targeted psychological approaches may be considered according to individual patient needs.

This study has several limitations. First, because of its single-center and cross-sectional design, causal relationships between variables cannot be established. FCR, anxiety, and depression were assessed using self-report scales, and no structured clinical interview was performed. Dichotomization of continuous scale scores may result in loss of statistical information and reduced statistical power; therefore, findings based on these binary classifications should be interpreted with this limitation in mind. In addition, although an FCRI-SF cut-off of ≥13 was used in accordance with the previous literature, higher cut-off values have also been proposed, and prevalence estimates may vary according to the threshold applied. In addition, several potentially relevant confounding factors, including marital and occupational status, social support, coping strategies, body image, financial toxicity, and family history of cancer, were not systematically evaluated. Although patients with active or severe psychiatric disorders that could interfere with participation or the reliability of scale responses were excluded, the omission of these additional psychosocial and sociodemographic factors may have resulted in residual confounding. The study population included a relatively high proportion of survivors with Stage III disease and a history of chemotherapy. Therefore, the findings may more strongly reflect the experiences of higher-risk and more intensively treated breast cancer survivors, and their generalizability to patients with earlier-stage disease or different treatment profiles may be limited. The inclusion of only patients who agreed to participate also means that the possibility of selection bias cannot be excluded. Although several associations were statistically significant, some effect estimates in the multivariable models were accompanied by relatively wide confidence intervals, indicating limited precision. Therefore, these findings should be interpreted with appropriate caution.

Despite these limitations, the study has several strengths. One important strength is the simultaneous evaluation of clinical, treatment-related, and psychological variables in a relatively homogeneous group of breast cancer survivors without evidence of active disease. The use of validated scales, identification of independently associated factors through multivariable analyses, and the real-world oncology practice setting further strengthen the methodology of the study. In addition, this study contributes to the limited body of evidence from Türkiye evaluating FCR together with psychological symptom burden among breast cancer survivors.

## 5. Conclusions

FCR emerges as an important psychosocial problem among breast cancer survivors without evidence of active disease. Younger age, chemotherapy history, and clinically significant psychological symptom burden were consistently associated with clinically significant FCR across two separate multivariable models. Mastectomy was also independently associated with FCR in the model including depressive symptoms. These findings indicate that routine oncology follow-up should systematically address FCR and related psychological symptom burden in addition to physical recurrence assessment. These findings support consideration of FCR and psychological symptom assessment during routine survivorship follow-up, particularly among patient groups showing stronger associations with clinically significant FCR. Given the cross-sectional nature of the study, these findings should be interpreted as associations rather than evidence of causal relationships.

## Figures and Tables

**Table 1:** Baseline demographic, clinicopathological, and treatment-related characteristics of the study population.

Variable	Overall (N = 200)
Age, years, mean ± SD	55.60 ± 9.51
Age, years, median (range)	56 (38–73)
Remission duration, months, median (range)	24 (6–96)
Menopausal status	
Pre-/perimenopausal	44 (22.0)
Postmenopausal	156 (78.0)
Disease stage at diagnosis	
Stage I	54 (27.0)
Stage II	32 (16.0)
Stage III	114 (57.0)
Educational level	
Illiterate/primary school	139 (69.5)
High school/university	61 (30.5)
Income status	
Income lower than expenses	69 (34.5)
Income equal to or higher than expenses	131 (65.5)
Smoking history	
No	160 (80.0)
Yes	40 (20.0)
Molecular subtype	
HR-positive/HER2-negative	122 (61.0)
HER2-positive	54 (27.0)
Triple-negative	24 (12.0)
Type of surgery	
Breast-conserving surgery	128 (64.0)
Mastectomy	72 (36.0)
History of chemotherapy	
No	60 (30.0)
Yes	140 (70.0)
History of radiotherapy	
No	47 (23.5)
Yes	153 (76.5)

Data are presented as mean ± standard deviation, median (range), or n (%), as appropriate. Abbreviations: HER2, human epidermal growth factor receptor 2; HR, hormone receptor; SD, standard deviation.

**Table 2:** Fear of cancer recurrence, anxiety, and depression characteristics.

Variable	Overall (N = 200)
Clinically significant FCR	
No, FCRI-SF < 13	124 (62.0)
Yes, FCRI-SF ≥ 13	76 (38.0)
Clinically significant anxiety symptoms	
No, HADS-A < 8	135 (67.5)
Yes, HADS-A ≥ 8	65 (32.5)
Clinically significant depression symptoms	
No, HADS-D < 8	124 (62.0)
Yes, HADS-D ≥ 8	76 (38.0)

Data are presented as n (%). Abbreviations: FCR, fear of cancer recurrence; FCRI-SF, Fear of Cancer Recurrence Inventory–Short Form; HADS-A, Hospital Anxiety and Depression Scale-Anxiety subscale; HADS-D, Hospital Anxiety and Depression Scale-Depression subscale.

**Table 3:** Comparison of patient characteristics according to clinically significant FCR status.

Variable	No FCR (N = 124)	FCR (N = 76)	*p* Value
Menopausal status			<0.001
Pre-/perimenopausal	7 (15.9)	37 (84.1)	
Postmenopausal	117 (75.0)	39 (25.0)	
Disease stage at diagnosis			<0.001
Stage I	46 (85.2)	8 (14.8)	
Stage II	24 (75.0)	8 (25.0)	
Stage III	54 (47.4)	60 (52.6)	
Remission duration, months, median (range)	24 (8–96)	24 (6–96)	0.292
Educational level			0.127
Illiterate/primary school	91 (65.5)	48 (34.5)	
High school/university	33 (54.1)	28 (45.9)	
Income status			0.247
Income lower than expenses	39 (56.5)	30 (43.5)	
Income equal to or higher than expenses	85 (64.9)	46 (35.1)	
Molecular subtype			0.148
HR-positive/HER2-negative	82 (67.2)	40 (32.8)	
HER2-positive	30 (55.6)	24 (44.4)	
Triple-negative	12 (50.0)	12 (50.0)	
Type of surgery			0.009
Breast-conserving surgery	88 (68.8)	40 (31.2)	
Mastectomy	36 (50.0)	36 (50.0)	
History of chemotherapy			0.031
No	44 (73.3)	16 (26.7)	
Yes	80 (57.1)	60 (42.9)	
History of radiotherapy			0.523
No	31 (66.0)	16 (34.0)	
Yes	93 (60.8)	60 (39.2)	
Clinically significant anxiety symptoms			<0.001
No, HADS-A < 8	101 (74.8)	34 (25.2)	
Yes, HADS-A ≥ 8	23 (35.4)	42 (64.6)	
Clinically significant depression symptoms			<0.001
No, HADS-D < 8	92 (74.2)	32 (25.8)	
Yes, HADS-D ≥ 8	32 (42.1)	44 (57.9)	
Smoking history			0.308
No	102 (63.8)	58 (36.2)	
Yes	22 (55.0)	18 (45.0)	

Abbreviations: FCR, fear of cancer recurrence; HADS-A, Hospital Anxiety and Depression Scale-Anxiety subscale; HADS-D, Hospital Anxiety and Depression Scale-Depression subscale; HER2, human epidermal growth factor receptor 2; HR, hormone receptor.

**Table 4:** Univariable logistic regression analyses of factors associated with clinically significant FCR.

Variable	Univariable OR	95% CI	*p* Value
Age, per 1-year increase	0.864	0.827–0.903	<0.001
Postmenopausal vs. pre-/perimenopausal	0.063	0.026–0.154	<0.001
Disease stage at diagnosis			<0.001
Stage II vs. Stage I	1.917	0.640–5.742	0.245
Stage III vs. Stage I	6.389	2.769–14.740	<0.001
High school/university vs. illiterate/primary school	1.609	0.871–2.970	0.129
Income equal to or higher than expenses vs. lower than expenses	0.704	0.388–1.277	0.248
Molecular subtype			0.151
HER2-positive vs. HR-positive/HER2-negative	1.640	0.851–3.162	0.140
Triple-negative vs. HR-positive/HER2-negative	2.050	0.846–4.967	0.112
Mastectomy vs. breast-conserving surgery	2.200	1.214–3.986	0.009
Chemotherapy: yes vs. no	2.062	1.063–4.002	0.032
Radiotherapy: yes vs. no	1.250	0.630–2.480	0.523
Remission duration, per-month increase	0.917	0.770–1.091	0.327
Clinically significant anxiety symptoms, HADS-A ≥ 8 vs. <8	5.425	2.861–10.287	<0.001
Clinically significant depression symptoms, HADS-D ≥ 8 vs. <8	3.953	2.153–7.258	<0.001
Smoking history: yes vs. no	1.439	0.714–2.902	0.309

Abbreviations: CI, confidence interval; FCR, fear of cancer recurrence; HADS-A, Hospital Anxiety and Depression Scale-Anxiety subscale; HADS-D, Hospital Anxiety and Depression Scale-Depression subscale; HER2, human epidermal growth factor receptor 2; HR, hormone receptor; OR, odds ratio; FCRI-SF, Fear of Cancer Recurrence Inventory–Short Form. Note: Clinically significant FCR was defined as FCRI-SF ≥ 13.

**Table 5:** Multivariable logistic regression Model 1 including HADS-A.

Variable	Adjusted OR	95% CI	*p* Value
Age, per 1-year increase	0.855	0.807–0.906	<0.001
Disease stage at diagnosis			0.088
Stage II vs. Stage I	0.317	0.062–1.620	0.167
Stage III vs. Stage I	1.453	0.478–4.417	0.510
Mastectomy vs. breast-conserving surgery	1.513	0.622–3.682	0.361
Chemotherapy: yes vs. no	5.841	1.680–20.309	0.006
Clinically significant anxiety symptoms, HADS-A ≥ 8 vs. <8	7.828	3.200–19.154	<0.001

Abbreviations: CI, confidence interval; FCR, fear of cancer recurrence; FCRI-SF, Fear of Cancer Recurrence Inventory–Short Form; HADS-A, Hospital Anxiety and Depression Scale-Anxiety subscale; HADS-D, Hospital Anxiety and Depression Scale-Depression subscale; OR, odds ratio. Note: Menopausal status was evaluated in univariable analysis but was not included in the multivariable models because of its close clinical and statistical relationship with age.

**Table 6:** Multivariable logistic regression Model 2 including HADS-D.

Variable	Adjusted OR	95% CI	*p* Value
Age, per 1-year increase	0.836	0.787–0.887	<0.001
Disease stage at diagnosis			0.704
Stage II vs. Stage I	0.804	0.207–3.120	0.753
Stage III vs. Stage I	0.590	0.169–2.060	0.408
Mastectomy vs. breast-conserving surgery	6.265	1.987–19.749	0.002
Chemotherapy: yes vs. no	6.941	2.109–22.847	0.001
Clinically significant depression symptoms, HADS-D ≥ 8 vs. <8	14.343	4.398–46.779	<0.001

Abbreviations: CI, confidence interval; FCR, fear of cancer recurrence; FCRI-SF, Fear of Cancer Recurrence Inventory–Short Form; HADS-A, Hospital Anxiety and Depression Scale-Anxiety subscale; HADS-D, Hospital Anxiety and Depression Scale-Depression subscale; OR, odds ratio. Note: Menopausal status was evaluated in univariable analysis but was not included in the multivariable models because of its close clinical and statistical relationship with age.

## Data Availability

The individual-level dataset cannot be publicly shared because of participant confidentiality, the scope of the informed consent obtained, and institutional ethical restrictions. Summary statistical outputs supporting the main findings of the study are available from the corresponding author upon reasonable request.

## Supplementary Materials

The following supporting information can be downloaded at https://www.mdpi.com/article/10.3390/healthcare14162665/s1. Table S1. Sensitivity multivariable logistic regression Model 1 including HADS-A and menopausal status; Table S2. Sensitivity multivariable logistic regression Model 2 including HADS-D and menopausal status.

## Abbreviations

FCR	Fear of cancer recurrence
FCRI-SF	Fear of Cancer Recurrence Inventory–Short Form
HADS	Hospital Anxiety and Depression Scale
HADS-A	Hospital Anxiety and Depression Scale–Anxiety subscale
HADS-D	Hospital Anxiety and Depression Scale–Depression subscale
HER2	Human epidermal growth factor receptor 2
HR	Hormone receptor
OR	Odds ratio
CI	Confidence interval
VIF	Variance inflation factor
SD	Standard deviation
