# Supplementary material for: Fear of Cancer Recurrence and Psychological Symptom Burden in Breast Cancer Survivors Without Evidence of Active Disease: A Cross-Sectional Real-World Study
Source: Healthcare (Basel). 2026 Aug 21;14(16):2665. doi: 10.3390/healthcare14162665 (PMC13511790; doi:10.3390/healthcare14162665)
Supplement: Supplementary file 1 [file healthcare-14-02665-s001.zip › healthcare-4498161-supplementary.pdf]

Supplementary Table S1. Sensitivity multivariable logistic regression Model 1 including HADS-A and menopausal status

Variable	Adjusted OR	95% CI	p value
Disease stage at diagnosis			0.046
Stage II vs Stage I	0.405	0.094–1.747	0.226
Stage III vs Stage I	1.770	0.630–4.975	0.279
Mastectomy vs breast-conserving surgery	1.676	0.746–3.766	0.211
Chemotherapy: yes vs no	3.341	1.249–8.940	0.016
Postmenopausal vs pre-/perimenopausal	0.083	0.029–0.237	<0.001
Clinically significant anxiety symptoms, HADS-A ≥8 vs <8	4.878	2.197–10.827	<0.001

Abbreviations: CI, confidence interval; FCR, fear of cancer recurrence; HADS-A, Hospital Anxiety and Depression Scale–Anxiety subscale; OR, odds ratio. Note: This sensitivity analysis repeated Model 1 by replacing age with menopausal status.

Supplementary Table S2. Sensitivity multivariable logistic regression Model 2 including HADS-D and menopausal status

Variable	Adjusted OR	95% CI	p value
Disease stage at diagnosis			0.596
Stage II vs Stage I	0.530	0.137–2.052	0.358
Stage III vs Stage I	0.900	0.303–2.674	0.850
Mastectomy vs breast-conserving surgery	4.496	1.657–12.198	0.003
Chemotherapy: yes vs no	4.423	1.582–12.372	0.005
Postmenopausal vs pre-/perimenopausal	0.049	0.016–0.149	<0.001
Clinically significant depression symptoms, HADS-D ≥8 vs <8	7.541	2.849–19.961	<0.001

Abbreviations: CI, confidence interval; FCR, fear of cancer recurrence; HADS-D, Hospital Anxiety and Depression Scale–Depression subscale; OR, odds ratio. Note: This sensitivity analysis repeated Model 2 by replacing age with menopausal status.
